# Subtotal Cholecystectomy for Intraoperatively Diagnosed Mirizzi Syndrome: A Case Report

**DOI:** 10.7759/cureus.114639

**Published:** 2026-08-17

**Authors:** Salwan K Alobaidi

**Affiliations:** 1 General/GI Surgery, Paky Hospital, Erbil, IRQ

**Keywords:** intraoperative diagnosis, laparoscopic partial cholecystectomy, mirizzi's syndrome, safe surgery, specialized center

## Abstract

Mirizzi syndrome is an infrequent yet intricate biliary pathology defined by extrinsic compression of the common hepatic duct, typically secondary to stone impaction within the cystic duct or Hartmann’s pouch. We describe the case of a 69-year-old male who presented in December 2025 with a protracted history of recurrent right upper quadrant abdominal pain, accompanied by a 10-day history of persistent vomiting and fever. Clinical and radiological investigations indicated severe gallbladder inflammation, raising suspicion of Mirizzi syndrome. The patient underwent successful laparoscopic partial cholecystectomy and subsequently experienced an uncomplicated recovery. He continues to be monitored in the outpatient setting. This case emphasizes the clinical viability of partial cholecystectomy as a secure surgical intervention when inflammatory adhesions preclude total gallbladder resection, thereby minimizing the risk of iatrogenic biliary injury.

## Introduction

This case report illustrates the use of subtotal cholecystectomy in the setting of severe gallbladder inflammation and dense pericholecystic adhesions, in which distorted anatomy increases the risk of iatrogenic biliary injury. In such circumstances, subtotal cholecystectomy provides a pragmatic and safer alternative to total cholecystectomy by preserving a gallbladder remnant and avoiding hazardous dissection near critical biliary structures.

Mirizzi syndrome is a rare and diagnostically challenging complication of cholelithiasis, occurring in approximately 0.7%-1.4% of patients undergoing biliary surgery. It results from the impaction of a gallstone, which causes extrinsic compression of the common hepatic duct and may range from simple external compression to advanced disease with cholecystobiliary fistula formation.

Mirizzi syndrome remains a complex complication of cholelithiasis, often presenting diagnostic difficulties that necessitate careful intraoperative decision-making [1]. This report details a case where the unexpected identification of biliary obstruction required a subtotal reconstructive approach to ensure patient safety while avoiding iatrogenic injury to the hepatic duct [2]. This clinical presentation underscores the utility of subtotal cholecystectomy as a prudent surgical strategy when the anatomical relationship between the gallbladder and the common hepatic duct is severely distorted by impacted calculi.

## Case presentation

A 69-year-old male presented to our department with a primary complaint of recurrent right upper quadrant abdominal pain, accompanied by a 10-day history of persistent vomiting and fever. His medical history was significant for hypertension, diabetes mellitus managed with oral hypoglycemic agents, and a prior laparotomy 20 years earlier due to a complicated duodenal ulcer. Physical examination revealed mild dehydration, right upper quadrant tenderness, and a palpable mass. Baseline laboratory investigations demonstrated normal hepatic markers. However, other findings indicated renal impairment, likely reflecting prerenal azotemia secondary to vomiting, as well as anemia and a pronounced inflammatory response, as summarized in Table 1.

**Table 1:** Laboratory results.

Laboratory parameter	Result	Normal range
Hemoglobin	10.8 g/dL	13.5-17.5 g/dL
White blood cell count	15.2 × 10⁹/L	4.0-11.0 × 10⁹/L
C-reactive protein	145 mg/L	<5 mg/L
Creatinine	1.6 mg/dL	0.7-1.3 mg/dL
Blood urea nitrogen	25 mg/Dl	7-20 mg/dL
Aspartate aminotransferase	32 U/L	10-40 U/L
Alanine aminotransferase	35 U/L	7-56 U/L
Total bilirubin	0.8 mg/dL	0.2-1.2 mg/dL

**Figure 1:**
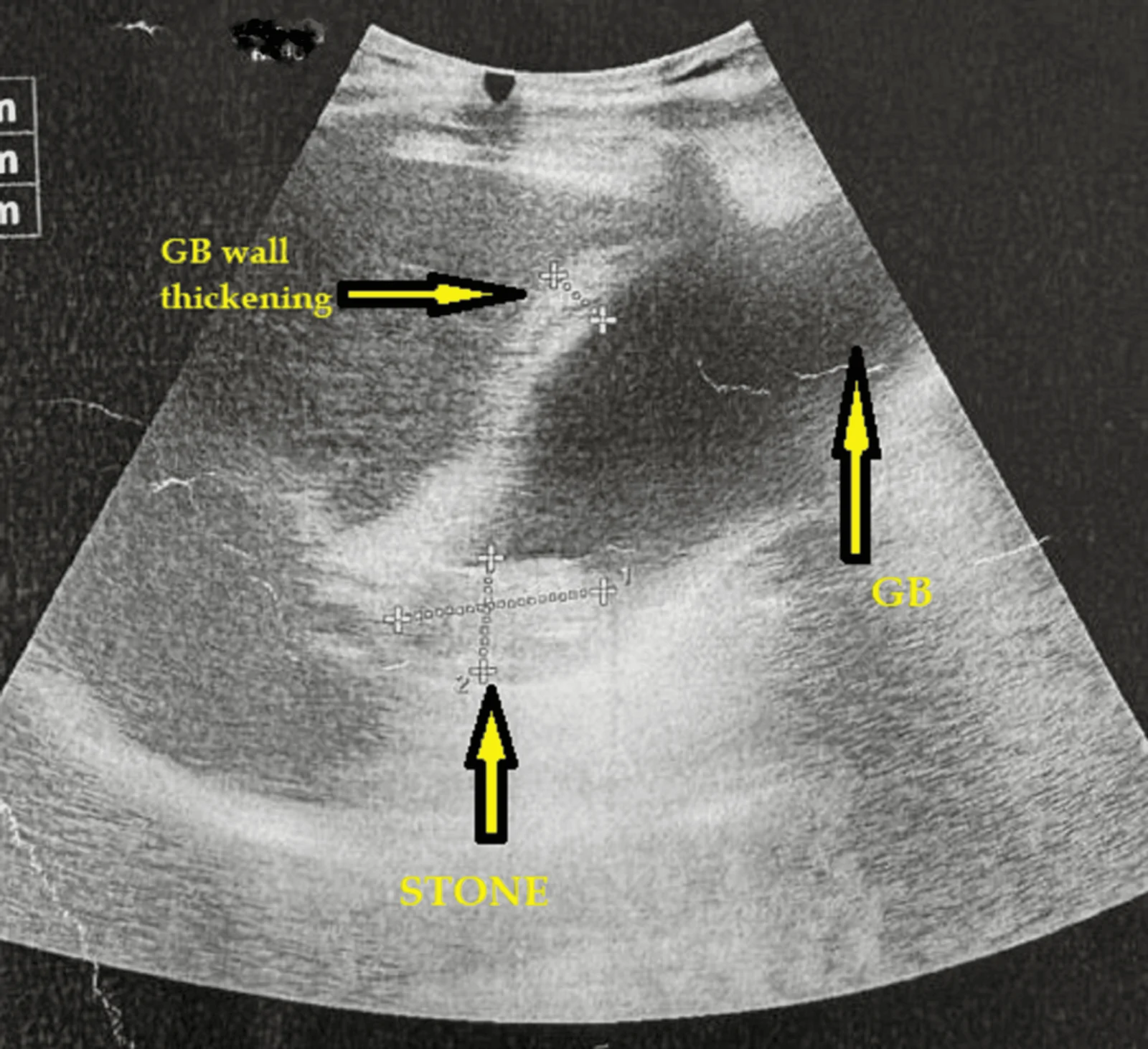
Ultrasonography showing gallbladder (GB) distension, wall thickening, and a stone at the GB neck.

Ultrasonographic evaluation revealed a distended gallbladder characterized by an irregular wall, multiple calculi, and pericholecystic fluid, in addition to a 30 × 23 mm nodular lesion at the gallbladder neck and proximal common bile duct dilation (Figure 1).

Contrast-enhanced computed tomography confirmed gallbladder distension with wall thickening and enhancement, as well as the presence of pneumobilia; no distinct mass was visualized, although biliary dilation persisted.

**Figure 2:**
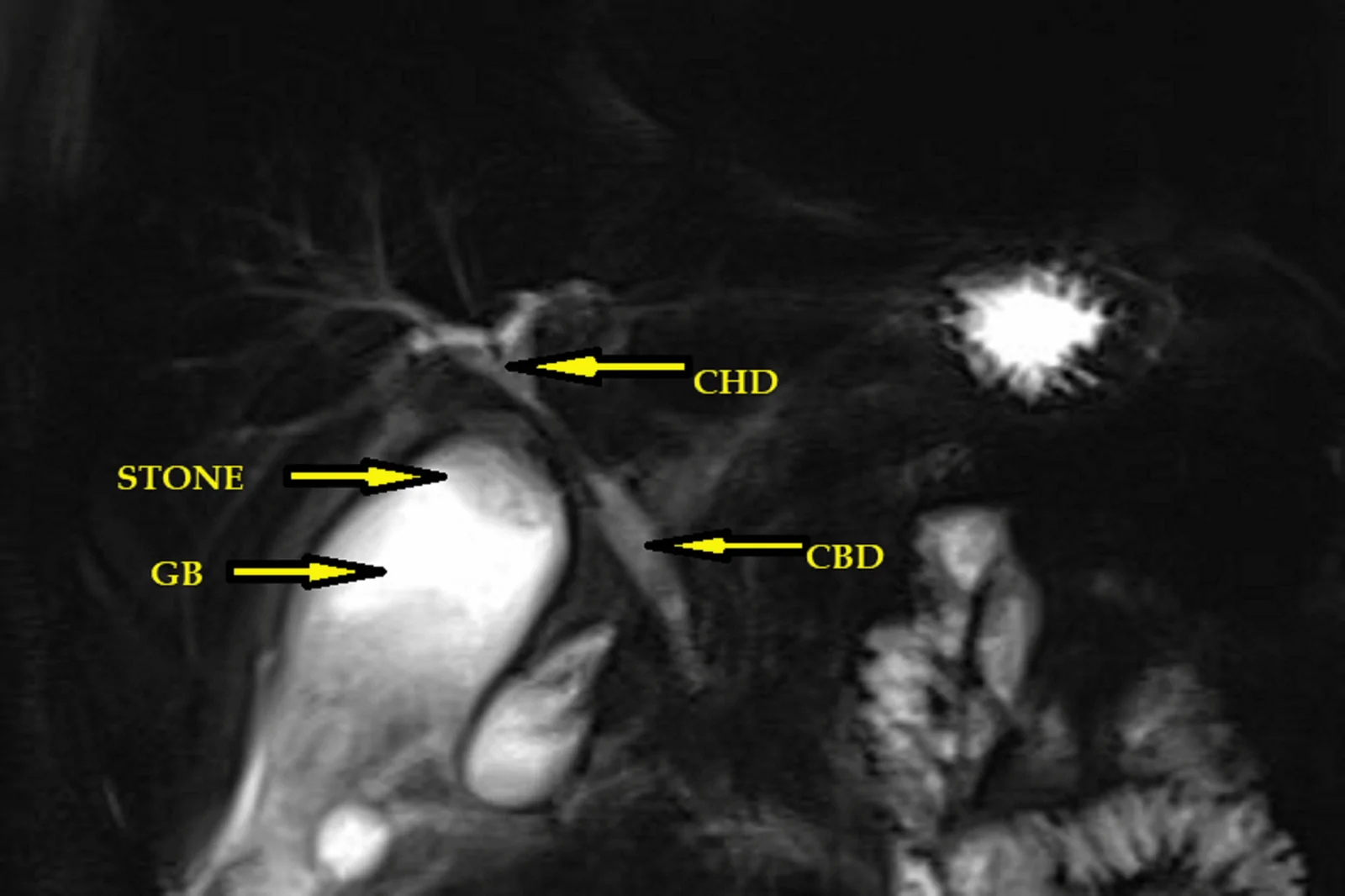
MRI showing the gallbladder (GB), stone, common hepatic duct (CHD), and common bile duct (CBD).

Magnetic resonance cholangiopancreatography demonstrated a distended gallbladder with diffuse wall thickening, a pericholecystic collection, and a 25 mm × 22 mm stone impacted at the neck. The associated dilation of intrahepatic ducts, attributed to extrinsic compression of the common hepatic duct by the gallbladder, raised strong suspicion of Mirizzi syndrome (Figure 2).

**Figure 3:**
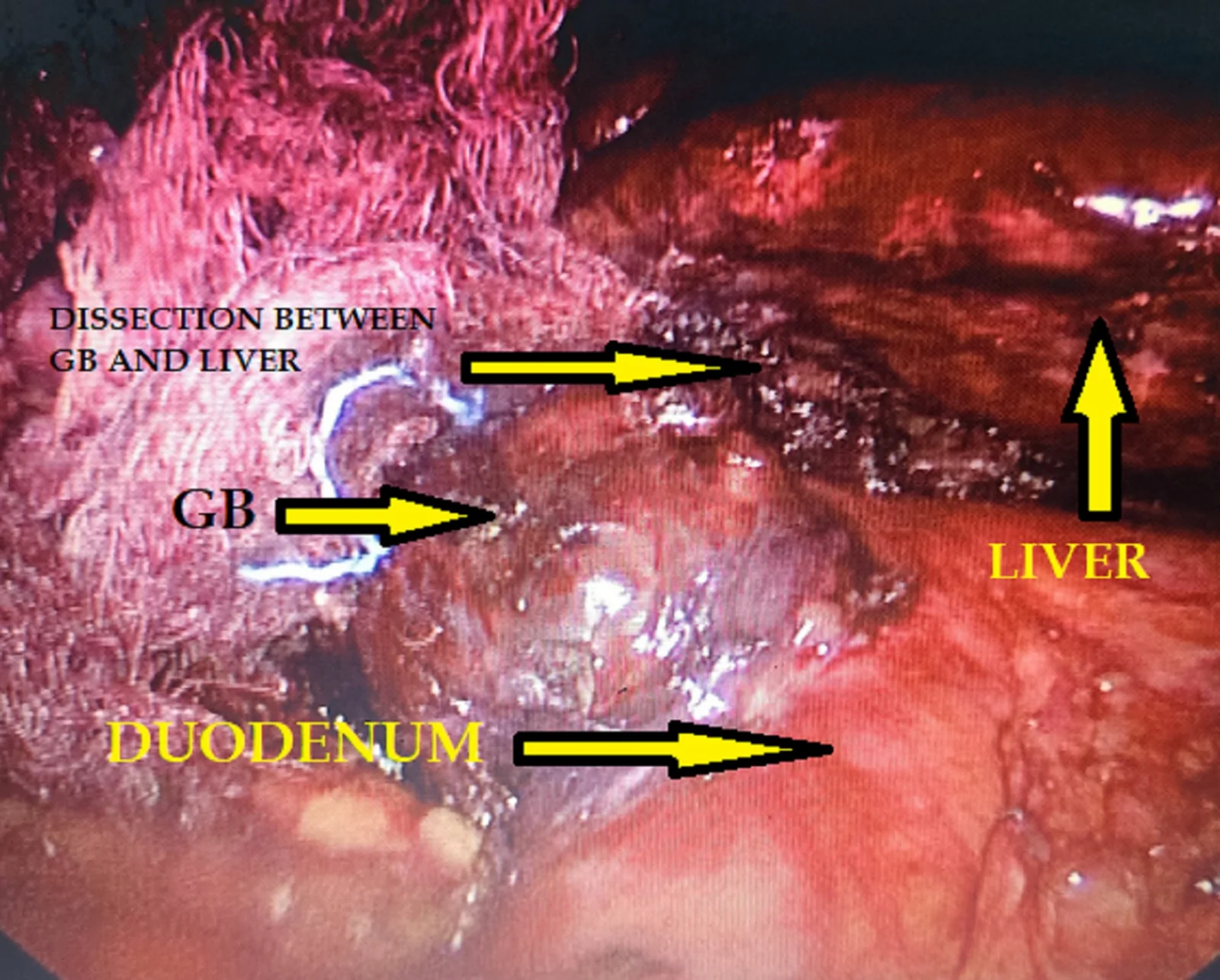
Intraoperative photograph showing the gallbladder (GB), duodenum severely adherent to the GB wall, liver, and the site of dissection between the GB and liver.

**Figure 4:**
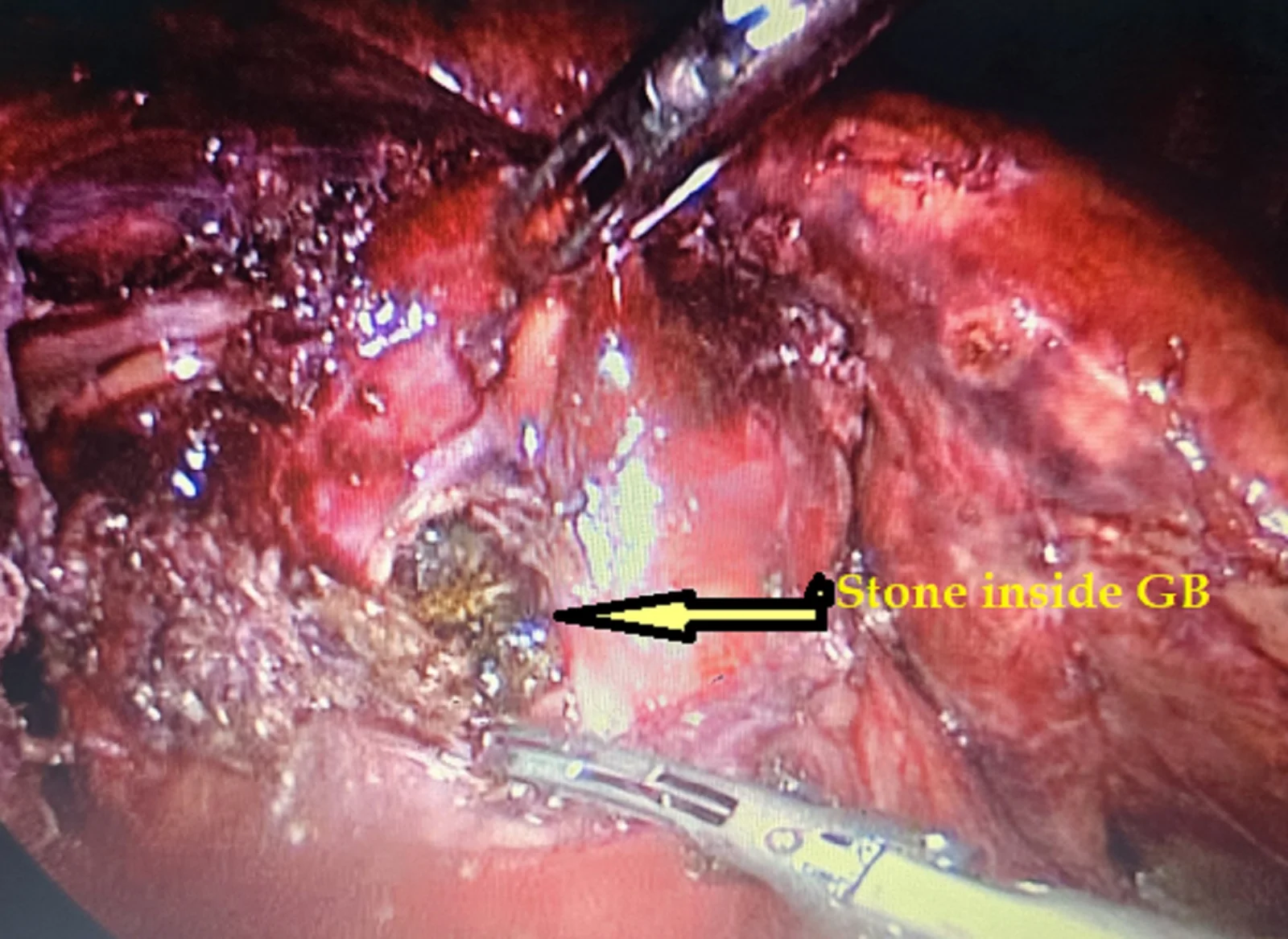
Intraoperative photograph showing a stone within the gallbladder (GB) after opening the GB wall.

**Figure 5:**
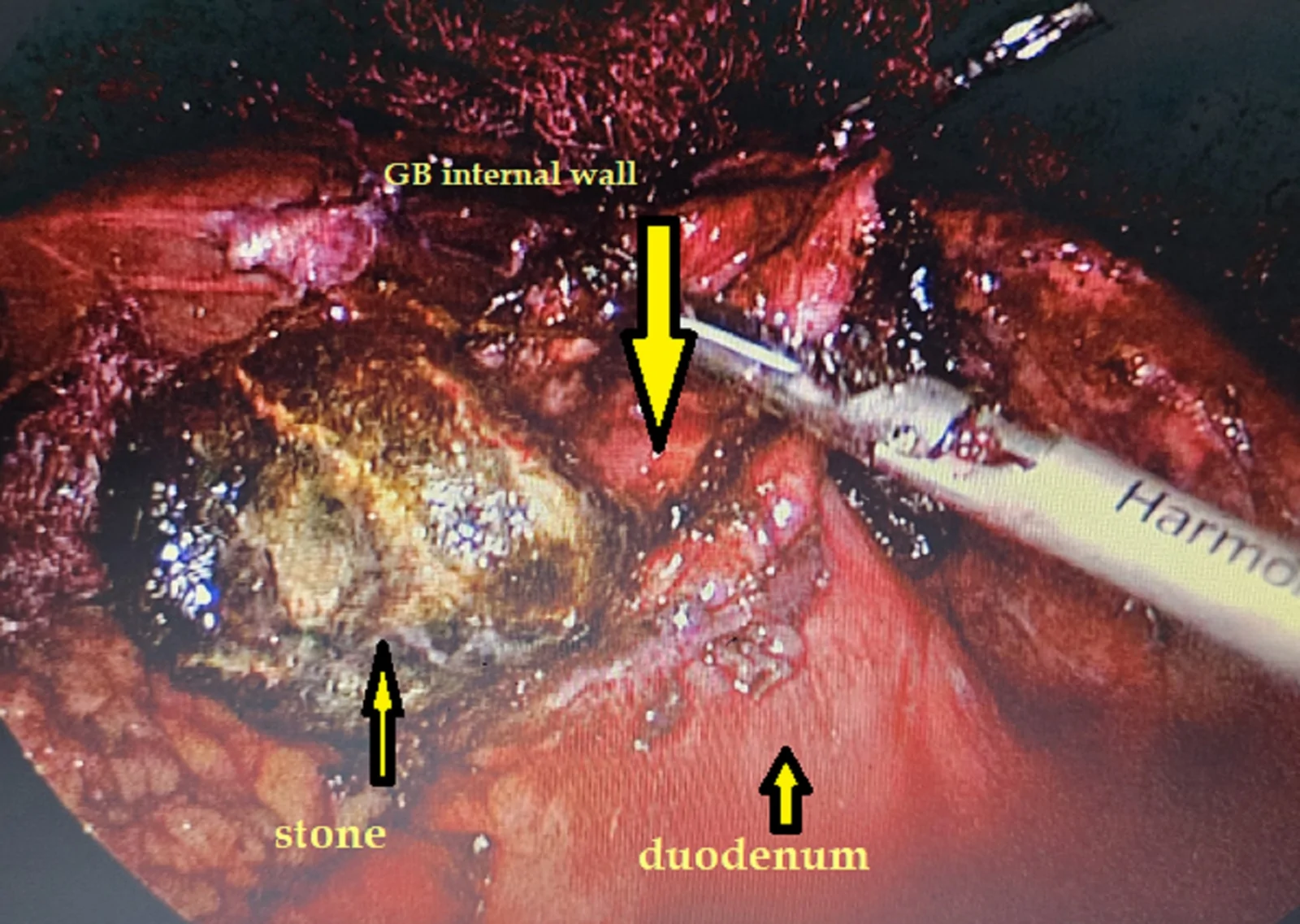
A large stone outside the gallbladder (GB) lumen, with the duodenum severely adherent to the GB wall.

Given the acute inflammatory status and high index of clinical suspicion for Mirizzi syndrome, the patient underwent laparoscopic exploration. Severe pericholecystic adhesions and intense inflammation in the region of Calot’s triangle necessitated a conversion to a subtotal, partial cholecystectomy to safeguard biliary integrity (Figures 3-5).

Following the extraction of the large, impacted calculus from the pus-filled gallbladder, a communication defect with the common hepatic duct was observed. To mitigate the risk of ductal trauma, the gallbladder remnant was managed via primary closure over a drain, facilitating adequate decompression and secure healing of the biliary tree. Postoperative recovery was uneventful. The patient’s inflammatory markers progressively declined, renal function normalized, fever resolved, and his overall clinical condition improved. He was discharged on postoperative day five with the drain in situ. The drain was subsequently removed during an outpatient follow-up appointment two weeks later, after the absence of biliary leakage had been confirmed. At six-month follow-up, his symptoms had completely resolved, and laboratory test results were within normal limits.

## Discussion

Preoperative diagnosis of Mirizzi syndrome remains inherently complex, primarily because the condition frequently mimics malignant biliary obstruction [1]. The condition may therefore be overlooked in patients presenting with persistent right upper quadrant pain, fever, or biliary dilatation. Diagnostic uncertainty is compounded by the similarity between its radiological features and those of malignant biliary obstruction, as imaging may not reliably distinguish extrinsic compression by an impacted gallstone from a primary biliary or gallbladder neoplasm. These limitations underscore the importance of maintaining a high index of clinical suspicion and correlating the patient’s clinical, laboratory, and radiological findings. This diagnostic challenge is particularly pronounced in anicteric patients, in whom Mirizzi syndrome should be considered even in the absence of jaundice. Given these diagnostic ambiguities and the profound anatomical distortions precipitated by impacted calculi, surgical management requires a flexible approach to mitigate the risk of iatrogenic biliary injury [2]. In such complex scenarios, subtotal cholecystectomy offers a definitive alternative to total resection, preventing inadvertent common bile duct injury when severe inflammation obscures critical anatomical landmarks [3,4]. By deliberately retaining the posterior gallbladder wall, the surgeon can circumvent hazardous dissection at the junction of the cystic and common hepatic ducts [5]. Due to the technical demands posed by distorted biliary anatomy, this procedure requires surgeons with specialized expertise in complex laparoscopic hepatobiliary operations; such proficiency is essential for preserving vascular supply and structural integrity, thereby minimizing postoperative biliary leaks and long-term strictures [6,7]. Furthermore, this technique is associated with a more favorable recovery profile compared to the morbidity often linked with extensive radical dissection in high-risk inflammatory environments [8]. Employing an antegrade dissection technique, from the fundus toward the infundibulum, can further facilitate navigation through dense pericholecystic adhesions [9]. This approach prioritizes the *critical view of safety*, thereby reducing the risk of bile duct injury even when dense fibrosis prevents the standard identification of portal structures [10]. Evidence from various surgical cohorts suggests that employing laparoscopic subtotal cholecystectomy-whether leaving the gallbladder stump open or closed-provides a viable strategy for completing procedures where traditional resection carries significant risk [11]. This conservative surgical strategy is particularly advantageous in cases of a *frozen* Calot’s triangle, where chronic inflammation renders conventional isolation of the cystic duct and artery both hazardous and technically infeasible [12,13].

## Conclusions

Subtotal cholecystectomy serves as a vital salvage intervention for the management of Mirizzi syndrome, reconciling the imperative for definitive calculus removal with the necessity of preserving biliary structural integrity. Nevertheless, given the technical challenges inherent in such complex pathologies, clinicians lacking specialized hepatobiliary training should prioritize patient safety by pursuing timely specialist consultation or facilitating transfer to high-volume centers.
